# Water Quality Index and Human Health Risk Assessment of Drinking Water in Selected Urban Areas of a Mega City

**DOI:** 10.3390/toxics11070577

**Published:** 2023-07-02

**Authors:** Rab Nawaz, Iqra Nasim, Ali Irfan, Amjad Islam, Ayesha Naeem, Nadia Ghani, Muhammad Atif Irshad, Maria Latif, Badar Un Nisa, Riaz Ullah

**Affiliations:** 1 Department of Environmental Sciences, The University of Lahore, Lahore 54000, Pakistan; 2 Research and Knowledge Transfer, INTI International University, Putra Nilai 71800, Malaysia; 3 Department of Environmental Sciences, Lahore College for Women University, Lahore 54000, Pakistan; 4 Department of Chemistry, Government College University Faisalabad, Faisalabad 38000, Pakistan; raialiirfan@gmail.com; 5 College of Chemistry and Chemical Engineering, Shantou University, Shantou 515031, China; 6 Department of Chemistry, The University of Lahore, Sargodha 40100, Pakistan; 7 Department of Pharmacognosy, College of Pharmacy, King Saud University, Riyadh 11451, Saudi Arabia

**Keywords:** water quality, water contamination, heavy metals, arsenic, health risk assessment

## Abstract

The present study was conducted to evaluate the quality of drinking water and assess the potential health hazards due to water contaminants in selected urban areas of Lahore, Pakistan. Water samples were collected from ten sites and analyzed for different physico-chemical parameters including turbidity, color, pH, total dissolved solids (TDS), nitrates, fluoride, residual chlorine, and total hardness. Additionally, heavy metal (arsenic) and microbial parameters (*E. coli*) were also determined in the water samples. Drinking water quality evaluation indices, including the water quality index (WQI) for physico-chemical and biological parameters and human health risk assessment (HHRA) for heavy metal were estimated using the analytical results of the target parameters. It was found in most of the areas that the levels of arsenic, fluoride, TDS, and residual chlorine were higher than those recommended by the National Environmental Quality Standard (NEQS) and World Health Organization (WHO) guidelines. In addition to the physico-chemical parameters, microbial content (*E. coli*) was also found in the drinking water samples of the selected areas. Statistical analysis of the results indicated that levels of target parameters in drinking water samples are significantly different between sampling sites. The WQI for all physico-chemical and microbial parameters indicated that drinking water in most of the areas was unfit and unsuitable (WQI > 100) for drinking purposes except for the water of Bhatti Gate and Chota Gaon Shahdara with a WQI of 87 and 91, respectively. Drinking water in these areas had a very poor WQI rating. According to HHRA, drinking water from the selected sites was found to be of high risk to children and adults. The carcinogenic risk of arsenic indicated that all samples were of high risk to both adults and children (4.60 and 4.37 × 10^−3^, respectively). Regular monitoring of drinking water quality is essential, and proactive measures must be implemented to ensure the treatment and availability of safe drinking water in urban areas.

## 1. Introduction

The availability of safe drinking water is a fundamental requirement for maintaining good human health. Water, being a renewable resource, is an essential element for sustaining life on Earth, and it is abundant on the planet’s surface as the most plentiful compound [1]. Due to urbanization, climate change and an increasing population, water will become even more limited [2]. At the ground level and in sewage waters, industrial effluents are identified as significant pollutants. Industrial effluents refer to the wastewater or liquid waste generated from industrial processes. These effluents often contain various harmful substances such as heavy metals, chemicals, oils, and other pollutants that have damaging impacts on the environment as well as on human health [3,4,5]. When such effluents are directly discharged into ground or sewage waters without proper treatment, they can contaminate the water and pose a threat to human health and the environment. On the other hand, for the underground level, fertilizers and pesticides are the main pollutants as they dissolve in water and seep into groundwater [6,7]. Industries disregard the need for treatment and discharging effluents and waste into water bodies, causing severe harm to the environment and threatening the health of aquatic ecosystems. Globally, industries often release their effluents and waste directly into water bodies without undergoing any form of treatment or purification, leading to the contamination of groundwater by heavy metals and other pollutants. There are various treatment technologies such as nano-remediation, the advanced oxidation process, phytoremediation, etc., for the effective treatment of water bodies [8,9,10]. Water resources in developing countries, such as Pakistan, are facing contamination issues caused by numerous industrial as well as anthropogenic activities. The inadequate water supply compels people to rely on highly polluted sources such as boreholes and shallow wells for drinking water, which poses significant health risks. Moreover, these contaminated water sources are unsuitable for domestic usage, further exacerbating the challenges faced by communities in accessing safe and clean water [11]. According to the Pak-SECA (2006) Pakistan Strategic Country Environmental Assessment Report, in Pakistan 2 billion gallons of wastewater are discharged into water bodies every day [12].

In urban and rural areas, microbial contamination is one of the serious problems. Microbial contamination occurs when sewage leaks into water supply pipelines, leading to the mixing of sewage with the drinking water distribution system [13]. Contaminated water in developing countries can serve as a medium for the transmission of pathogens such as Rotavirus, Entamoeba histolytica, Campylobacter, E. coli, and other harmful micro-organisms, posing a risk of infection to humans [14,15,16]. Moreover, the presence of *E. coli* in water serves as a reliable indicator of potential contamination from sewage or animal waste, highlighting the need for thorough water quality assessments and appropriate remedial measures. It is estimated that about 2.3 billion public worldwide are suffering from waterborne diseases [17,18]. More than 2.2 million citizens die annually in developing countries because of a poor sanitation system and the consumption of contaminated water. Based on the Sustainable Development Goals (SDGs), approximately 1.2 billion individuals globally do not have access to even the most basic level of water services. It is noteworthy that eight out of ten people lack access to basic drinking water services in rural areas, with nearly half of them residing in the least developed countries (LDCs) [19]. Approximately 60% of the infant mortality rate is linked to parasitic water-borne diseases. About 90% of the population in the rural areas in Pakistan does not have access to safe drinking water [20]. According to a UNICEF report, about 20–40% of patients in Pakistan’s hospitals are suffering from water-related diseases, e.g., cholera, diarrhea, malaria, hepatitis, typhoid fever, dysentery, giardiasis and the rate of infant deaths and fertility is 12.6% and 7%, respectively. Due to diarrhea, about 0.2–0.25 million children in Pakistan pass away yearly. In Karachi, contaminated water causes a renal infection that causes 10,000 human deaths annually [21].

Drinking water that is polluted by tanneries’ effluents causes 82% of diseases such as typhoid, dysentery, and cholera because tanneries can significantly impact drinking water quality by introducing various pollutants into the environment. The primary concern arises from the discharge of untreated or poorly treated effluents containing harmful substances such as heavy metals, organic compounds, and high levels of salts into water bodies. These pollutants can infiltrate groundwater sources or contaminate surface water, posing risks to the quality and safety of drinking water supplies. The improper handling and disposal of tannery waste can lead to long-term environmental degradation and potential health hazards to communities using these water sources [22]. In the same way, the presence of chemicals and heavy metals such as nitrates, arsenic, fluorides, and lead in water bodies causes many health problems [23,24,25]. Heavy metals present in water may cause higher levels of pathogenicity in some bacterial species [26,27]. According to an official survey from 12 districts of Punjab, about 79% of drinking water samples were found to be contaminated while in the rural areas, 88% of drinking water is polluted with fertilizers, heavy metals, microbes and sewage disposal [28]. Arsenic is indeed an important parameter to consider when assessing the quality of drinking water. Arsenic contamination of drinking water is a significant issue worldwide, affecting numerous regions and populations. It is particularly prevalent in certain parts of the world, such as Bangladesh, India, Nepal, Pakistan and various countries in Southeast Asia, where naturally occurring arsenic is present in groundwater sources. Due to the high toxicity, arsenic has severe health effects when consumed in elevated concentrations over a long period. Prolonged exposure to high levels of arsenic in drinking water has been linked to various health problems, including skin lesions, cancers (such as skin, lung, bladder, and kidney cancers), cardiovascular diseases, and neurological disorders [29]. Regular drinking water quality monitoring is essential to prevent an excessive amount of contamination [30]. With respect to Lahore, chlorination, regular monitoring and an increased number of water filtration plants can improve this situation [31].

To assess water quality, it is essential to have a clear understanding of how environmental characteristics are distributed spatially. However, monitoring water quality is often costly, especially for large groundwater basins. Therefore, reliable and adaptable devices are needed to address such issues. Technologies such as a geographic information system (GIS) can aid in addressing these issues by enabling spatial analysis, water quality monitoring, and assisting in strategic planning and decision-making processes related to water management. GIS can also aid in the real-time monitoring, tracking, and visualization of affected areas, populations at risk, and available resources in the case of waterborne disease outbreaks or emergencies. Interactive maps and visualizations created by GIS can help in raising awareness about water quality, sources, and potential risks and communicate water-related information to communities. By leveraging the power of GIS technology, fewer observations are needed to evaluate drinking water quality across the entire region, thereby reducing costs and enhancing the overall efficiency of water management and monitoring efforts. The water quality index and human health risk assessment are among the most commonly used tools for categorizing and reflecting the state of the water and the health risk in a certain area. Researchers from various nations have used WQI and HHRA to assess the water quality in diverse regions [32,33]. This study was conducted to assess the drinking water quality through the water quality index (WQI) and human health risk assessment (HHRA) in two age groups including adults and children. Additionally, the findings of the groundwater’s biochemical parameters were assessed by comparing them to national environmental quality standards (NEQS), which were then cross-validated by comparing them to the WHO’s groundwater parameters. Table 1 provides information on several chemical properties of groundwater properties [34].

## 2. Materials and Methods

**Figure 1:**
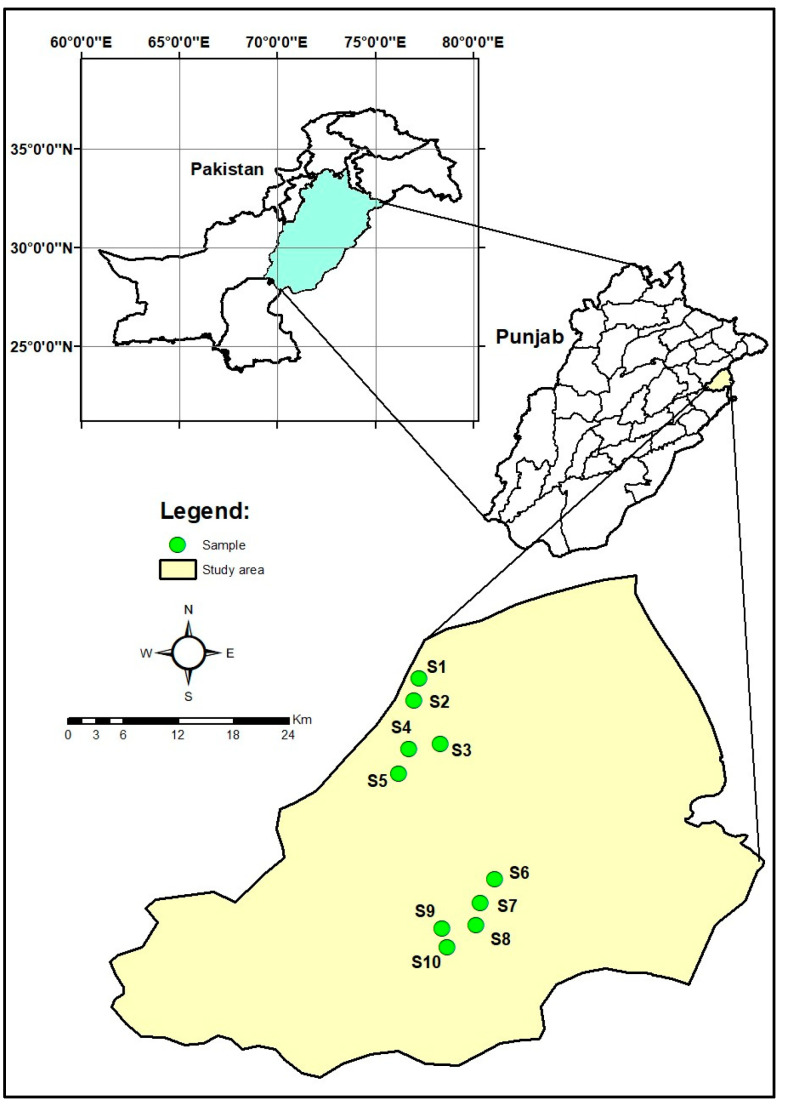
Study area map showing map of Pakistan, highlighting Punjab Province and Lahore district. Sampling sites (S1, S2, S3, S4, S5, S6, S7, S8, S9 and S10) are shown in selected urban areas of Lahore.

The present study was conducted in the densely populated city of Lahore of Punjab province, Pakistan. Lahore lies between a 31.5204° N latitude and 74.3587° E longitude, as shown in map (Figure 1) [35].

Samples of drinking water were collected from ten sampling sites in Lahore including Chota Gaon Shahdara (S1), Sant Nagar (S2), Bhutto Colony Shahdara (S3), Bhatti Gate (S4), Brendreth Road (S5), Nishtar Colony (S6), Gajjumatta (S7), Attari Saroba (S8), Tibba Kacha (S9) and Islampura (S10). Thirty (*n* = 30) water samples were collected from selected sites because these areas are less developed and highly populated. Water samples were collected using simple random sampling. Plastic and glass bottles were used for the collection of water samples (Table 2). These bottles were washed with distilled water before sampling. Water samples were collected from different water supplies including tap water, hand pumps, and motor pumps, these having groundwater. After sample collection, sampling bottles were sealed and labeled with details of the water source, location, sampling date and time.

### 2.1. Physico-Chemical and Biological Parameter Analysis

Physical parameters of water samples including temperature, color, pH and turbidity were measured at the place of sample collection. A pH meter was used to measure the pH of water samples. Some water sampling tests are mandatory for measuring the real physical properties of water quality [38]. A turbidity meter was used to measure the turbidity of water samples. All of these physico-chemical parameters that were examined using the standard methods described in Standard Methods for the Examination of Water and Wastewater [39].

Chemical parameters of water samples including fluoride, total hardness as CaCO_3_, nitrates (NO_3_^−^), residual chlorine and arsenic level were measured in the analytical laboratory. EDTA titration was used to measure the total hardness as CaCO_3_ in drinking water. A spectrophotometer was used to measure nitrates in the water samples [30]. A spectrophotometer was used to measure fluoride in water samples. For the determination of arsenic levels in water samples, an atomic absorption spectrophotometer (AAS) was used [40].

The biological parameter of water samples (*E. coli*) was measured via the membrane filtration method by using the Millipore membrane filtration system with a pore size of 0.45 μm and diameter of 47 mm. Drinking water samples were filtered using Millipore membrane filters, leaving micro-organisms on the surface of the filter. On the chromogen surface, these membrane filters were placed and incubated at 35 °C for 24 h. On the surface of the chromogen, colonies were formed. By using the colony counter, these colonies were counted [41].

### 2.2. Water Quality Index

Different WQI models have been created and used globally in recent years to assess the quality of surface and groundwater. The literature shows that the WQI model is one of the most efficient rating methods, which is used for scaling and quality assessment of water [42,43].

WQI was calculated by using the given weighted arithmetic index technique. The eight significant physiochemical parameters (turbidity, color, pH, TDS, nitrates, fluoride, residual chlorine and total hardness) were utilized regarding their appropriateness for human consumption. NEQS for drinking water were used to compare these parameters by using the formula for calculating WQI given by Tiwari et al., 2015 [44].

For WQI, firstly relative weight (*w_i_* (*W_i_*) was calculated using the following formula:

(1) $$w_{i}=\frac{K}{S_{i}}$$

“*K*” was calculated using the following equation;

(2) K=1(∑1Si)

where, “*W_i_*” is the unit weight factor, “*K*” is the proportional constant and “*S_i_*” is the standard permissible value of *i*th parameter.

The quality rating scale (*q_i_*) of ith parameter of contaminated water was calculated using the following equation:

(3) $$q_{i}=\frac{V_{i}}{S_{i}}\times 100$$

where, “*q_i_*” is the quality rating scale of ith parameter, “*V_i_*” is the mean concentration of *i*th parameter and “*S_i_*” is the standard permissible value of *i*th parameter.

So, after finding *w_i_* and *q_i_*, both values were multiplied by each other by having *w_i_q_i_*,

(4) wiqi=wi×qi

Then, overall *WQI* was calculated using the following equation;

(5) $$WQI=\sum_{i=1}^{n}w_{i}\times q_{i}$$

The quality of water based on a *WQI* may be categorized into five categories, including excellent, good, poor, very poor, and unfit, as shown in Table 3 [45].

### 2.3. Human Health Risk Assessment

The HHRA was classified as either carcinogenic or non-carcinogenic based on the evaluation of the risk level for exposure to metal or metalloids. The present study evaluated the rate of pollutant ingestion in a human body through the oral consumption of drinking water using the USEPA guidelines by computing the chronic daily intake (*CDI*), hazard quotient (HQ), hazard index (HI), and carcinogenic risk (CR).

#### 2.3.1. Hazard Quotient

The adverse effects resulting from exposure to non-carcinogenic pollutants were evaluated using a hazard quotient (HQ), as determined by the toxicant section of the United States Environmental Protection Agency (USEPA). The following equation was used to calculate HQ:

(6) $$HQ=\frac{CDI}{RfD}$$

The reference dosage (RfD) for arsenic is 0.0003 mg/kg/day [34].

The chronic daily intake (*CDI*) is computed using the following equation [46]:

(7) $$CDI=\frac{C\times IR\times EF\times ED}{BW\times AT}$$

where “*C*” is the concentration of heavy metals in the water (mg/L), “*IR*” is the rate at which people drink water (3.53 L/day for adults, 1.0 L/day for children, and 0.25 L/day for infants, according to [47]), “*ED*” is the duration of exposure in years (70 years for adults, 6 years for children, and 1 year for infants, according to [48]), and “*EF*” is the exposure frequency in days (d) (365 days for adults, children, and infants [46]). “*BW*” is the average body weight in kg (50 kg for adults, 15 kg for children, and 6.9 kg for infant [46,49]) and “*AT*” is the average time (*AT* = 365 × *ED*(d)).

#### 2.3.2. Hazard Index

The following equation is used to determine the HI of Arsenic associated with the HQ values, which is defined as the sum of the HQ of the investigated parameters.

(8) HI=HQ1+HQ2+HQ3+HQ4………HQn

HQ values show that there are no and considerable non-carcinogenic health impacts, respectively, if HQ < 1 and HQ > 1. HI < 1 denotes a minimal or non-existent risk of adverse non-cancer health, while HI > 1 denotes a high amount of such risk. There are four categories for chronic risk (HQ or HI): negligible (<0.1), low (≥0.1 < 1), medium (≥1 < 4), and high (≥4) [50].

#### 2.3.3. Carcinogenic Risk

By exposing people to carcinogenic substances, *CR* helps in estimating a person’s lifetime risk of developing any sort of cancer [46]. The slope factor (*SF*) of heavy metals produced by cancer is multiplied by the *CDI* to determine the cancer risk (*CR*), as shown in the following equation:

(9) CR=CDI×SF

where, “*SF*” (1.5 mg/kg/day for As) is the slope factor of pollutants [46]. The “CR” is identified from very low (<1 × 10^−6^) to very high (>1 × 10^−3^) [50].

### 2.4. Statistical Analysis

Water quality data were statistically analyzed by performing an analysis of variance (ANOVA) at *p* ≤ 0.05 and determining the least significant difference (LSD = 0.05). Significant difference was shown in the graphs using alphabetical letters (e.g., a, b, c) on the bars for different sampling sites. The NEQS and WHO guidelines were used in the graphs for comparison with the water quality parameters for the selected ten sites.

## 3. Results and Discussion

### 3.1. Physical Parameters

**Figure 2:**
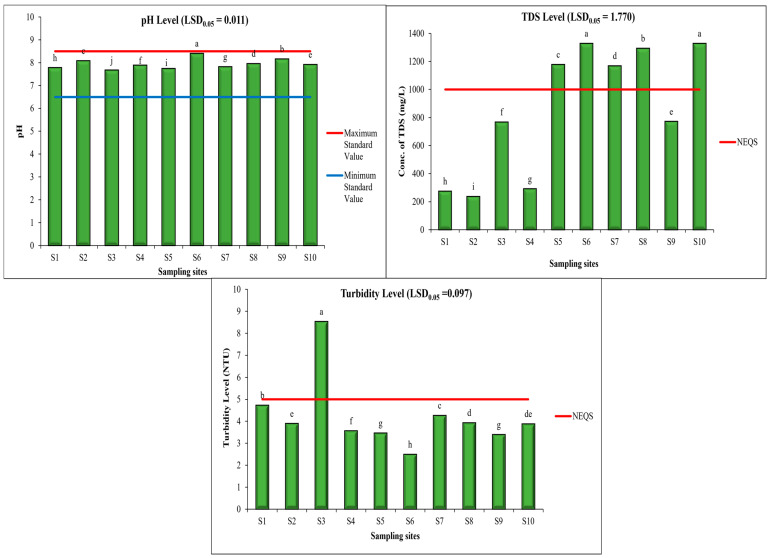
Levels of pH, Turbidity, and TDS in water samples in different areas of Lahore. Mean values are given in the graphs. Horizontal line shows standard value for the parameter. Letters (e.g., a, b, and c) show that values are significantly different.

The average pH of drinking water samples was found to be 8.004, with minimum and maximum levels of 7.75 and 8.40 at S5 and S6, respectively, as shown in Figure 2. However, it is noteworthy that the pH levels in all areas remained within the NEQS. ANOVA results indicated that in the pH levels of drinking water samples of sampling sites are significantly different. The following clusters were distinguished on the basis of the statistical analysis (LSD_0_._05_ = 0.011): a—8.40 (S6); b—8.16 (S9); c—8.09 (S2); d—7.96 (S8); e—7.92 (S10); f—7.89 (S4); g—7.82 (S7); h—7.78 (S1); i—7.75 (S5); and j—7.67 (S3). According to the NEQS, the pH of water ranges from 6.5–8.5. If the pH of water is above 8.5, it means it has a high level of alkalinity (Figure 2). The high level of alkalinity does not cause health problems but it can cause aesthetic harm such as a change in the flavor of the water, and the reduced efficiency of electric water heaters [51,52].

The average TDS value of drinking water samples was found to be 899.3 mg/L, with a minimum and maximum TDS value of 238 and 1329 mg/L at S2 and S10, respectively. ANOVA results indicated that the levels of TDS in drinking water samples of sampling sites are significantly different. The following clusters were distinguished on the basis of the statistical analysis (LSD_0_._05_ = 1.770): a-1328 (S6) and 1329 (S10); b-1294 (S8); c-1178 (S5); d-1169 (S7); e-772 (S9); f-768 (S3); g-292 (S4); h-275 (S1); and i-238 (S2). According to a study [53], TDS ranged from 675.3 to 1224 mg/L in Islampura, and was higher than the NEQS guideline. The high level of TDS is due to the presence of a higher level of inorganic compounds in water. In drinking water, higher levels of TDS cause scaling on water distribution pipelines and impart an undesirable flavor [54].

The average turbidity of drinking water samples was found to be 4.059 NTU, with the minimum level as 2.50 NTU and the maximum as 8.53 NTU at S6 and S3, respectively. ANOVA results indicated that the levels of turbidity in drinking water samples of sampling sites are significantly different. The following clusters were distinguished on the basis of the statistical analysis (LSD_0_._05_ = 0.097): a-8.53 (S3); b-4.73 (S1); c-4.27 (S7); d-3.94 (S8); e-3.90 (S2); de-3.88 (S10); f-3.57 (S4); g-3.47 (S5); 3.40 (S9); and h-2.50 (S6). It was found that the water at site S3 was highly turbid and hence, not safe for drinking purposes. The turbidity results of drinking water samples at S1, S2, S4 to S10 were within the NEQS guidelines and water was safe for drinking and other domestic purposes. Turbidity is caused by the existence of suspended and dissolved particles in water bodies that scatter light and make the appearance of water cloudy [55]. It can also increase the cost of the treatment of water for food processing and drinking purposes. Highly turbid water is also harmful to aquatic life such as fish because it affects gill function [56].

### 3.2. Chemical Parameters

**Figure 3:**
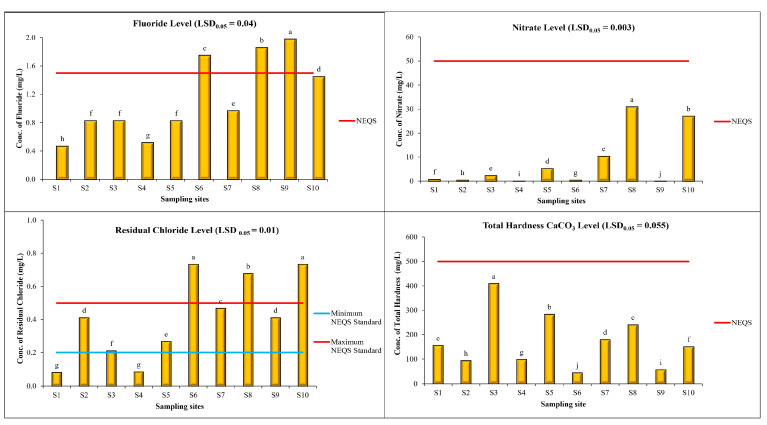
Levels of chemical parameters (nitrates, fluoride, residual chloride and total hardness CaCO_3_) in water samples in different areas of Lahore. Mean values are given in the graphs. Horizontal line shows standard value for the parameter. Letters (e.g., a, b, and c) show that values are significantly different.

The average level of fluoride in drinking water samples was found as 1.149 mg/L, with minimum and maximum levels of 0.47 and 1.98 mg/L at S1 and S9, respectively. ANOVA results indicate that level of fluoride in drinking water samples of sampling sites is significantly different. The following clusters were distinguished on the basis of the statistical analysis (LSD_0_._05_ = 0.04): a-1.98 (S9); b-1.86 (S8); c-1.75 (S6); d-1.45 (S10); e-0.97 (S7); f-0.83 (S2), 0.83 (S3) and 0.83 (S5); g-0.52 (S4) and h-0.47 (S1), respectively as shown in Figure 3. Fluoride is a naturally occurring element which is mostly found in drinking water and food [53]. Low to high concentrations of fluoride can be found in groundwater depending on the nature of the rock and minerals. Consequently, people living in hot climatic regions such as Lahore, particularly in the summers, are at risk of excessive fluoride intake due to their consumption of large volumes of water. An excessive concentration of fluoride causes skeletal fluorosis and also affects the kidneys and nervous system as well as causing dental fluorosis, bone deformation, and joint deformation [57]. The concentration of fluoride is increasing day by day due to human activities. If the concentration of fluoride is higher in water, it causes many health effects such as respiratory failure, anemia, weight loss, general paralysis, blood pressure, and bone deformation problems. It can also cause permanent inhibition of growth due to continuous ingestion. If the fluoride concentration in drinking water is below 0.8 mg/L, there is a need to take supplements for the proper growth of teeth in children. However, if the concentration of fluoride is 4–6 ppm in water, it causes dental and skeletal fluorosis [58].

The average nitrate value of drinking water samples was 7.77 mg/L, with a minimum nitrate level of 0.004 mg/L and a maximum nitrate level of 30.94 mg/L at S9 and S8, respectively. ANOVA results indicated that levels of nitrates in drinking water samples of sampling sites are significantly different. The following clusters were distinguished on the basis of the statistical analysis (LSD_0_._05_ = 0.003): a—30.94 (S8); b—27.03 (S10); c—10.38 (S7); d—5.26 (S5); e—2.32 (S3); f—0.77 (S1); g—0.51 (S6); h—0.49 (S2); i—0.02 (S4); and j—0.004 (S9), respectively. Nitrates occur naturally in water and soil and for plants they are a primary source of nitrogen [59]. Nitrate is not a concern at the natural level but a high level of nitrates causes many health problems in humans and livestock [60,61]. Nitrates drinking water bodies due to the widespread utilization of fertilizers in the agriculture sector and also due to municipal and industrial discharges and animal waste and can create the risk of cancers in human [62].

The average level of residual chlorine in drinking water samples was found to be 0.41 mg/L, with minimum and maximum levels of 0.08 and 0.73 mg/L at S1 and S10, respectively. ANOVA results indicated that the levels of residual chlorine in drinking water samples of sampling sites are significantly different. The following clusters were distinguished on the basis of the statistical analysis (LSD_0_._05_ = 0.01): a—0.73 (S6) and 0.73 (S10); b—0.68 (S8); c—0.47 (S7); d—0.41 (S2) and 0.41(S9); e—0.27 (S5); f—0.21 (S3); g—0.08 (S1); and 0.08 (S4). It should be noted that adherence to the specified residual chlorine concentrations is crucial for ensuring the safety and quality of water for consumption. Chlorine is a very harmful toxin but it is used to disinfect water to kill many harmful bacteria and micro-organisms and also to stop the growth of micro-organisms. Residual chlorine is much more toxic at a pH lower than 6.3. Within the body, chronic exposure to chlorine increases free radical production and causes cancer. Long-term exposure of children to chlorine causes asthmatic attacks. It can also damage the skin and cause irritation of the eyes and throat [63].

The average total hardness as CaCO_3_ value of drinking water samples was 171.18 mg/L, with a maximum level of 408.87 mg/L and minimum level of 44.82 mg/L at S3 and S6, respectively. ANOVA results indicated that levels of total hardness as CaCO_3_ in drinking water samples of sampling sites are significantly different. The following clusters were distinguished on the basis of the statistical analysis (LSD_0_._05_ = 0.055): a—408.87 (S3); b—283.17 (S5); c—240.54 (S8); d—179.38 (S7); e—155.70 (S1); f—150.83 (S10); g—98.48 (S4); h—93.36 (S2); i—56.69 (S9); and j—44.82 (S6). The possible reason for a high level of hardness is the presence of higher levels of limestone and carbonates, rocky material, marble industries, and inorganic materials. Water with a high content of minerals such as calcium, carbonates, bicarbonates, and CaCO_3_ is called hard water [11]. The hardness of water should be less than 500 mg/L per the WHO guidelines. The dissolved metallic polyvalent ions from the sedimentary rocks, runoff, and seepage from the soils are the natural sources of hardness in water bodies [64].

### 3.3. Biological Parameters

**Figure 4:**
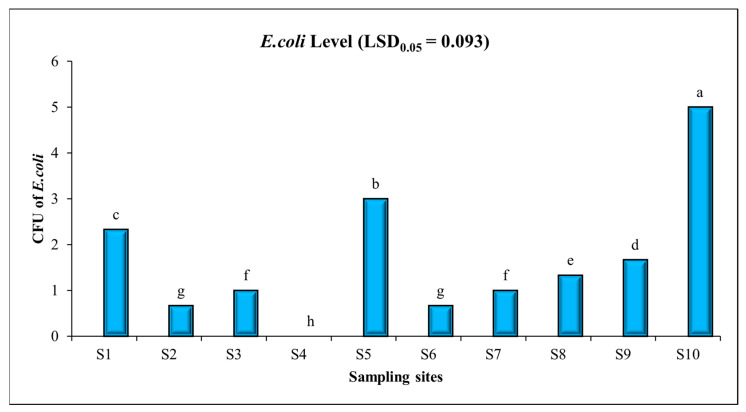
Level of biological parameter (*E. coli*) in water samples in different areas of Lahore. Mean values are given in the graph. Letters (e.g., a, b, and c) show that values are significantly different.

The average *E.coli* level of drinking water samples was found to be 1.67, with minimum and maximum levels of 0.00 and 5.00 at S4 and S10, respectively. Per NEQS, the presence of *E. coli* should be 0 colony forming units (CFU) per 100 mL of the water sample. ANOVA results indicated that the levels of *E. coli* in drinking water samples of sampling sites are significantly different. The following clusters were distinguished on the basis of the statistical analysis (LSD_0_._05_ = 0.093): a—5.00 (S10); b—3.00 (S5); c—2.33 (S1); d—1.67 (S9); e—1.33 (S8); f—1.00 (S3), 1.00 (S7); g—0.67 (S2); 0.67 (S6); and h—0.00 (S4), as shown in Figure 4. In the study area, the pipes used for water distribution were primarily made of galvanized iron and asbestos cement, with an age ranging from 30 to 35 years. It was observed that in certain areas, water supply and sewer lines were laid in close proximity to each other, without taking into account the necessary safe distance between the two lines. This particular situation has contributed to the bacteriological contamination found in the water distribution system. If *E. coli* is present in the water, is the latter is bacteriologically contaminated. Chronic exposure to bacteriologically contaminated water infects the lungs, skin, eyes, kidneys, liver, and nervous system [65,66]. A study conducted in the Vehari district of Punjab province revealed the presence of *E. coli* and coliforms in drinking water, indicating non-compliance with WHO guidelines [67]. A high bacterial count in 1066/0.5 mL of drinking water was observed in other study [68]. It was also found that 84.5% of the samples were contaminated with *E. coli* and coliforms, highlighting the unsatisfactory state of drinking water supplies for the public and a deteriorating water quality trend [69]. These findings align with those of previous reports from various locations in Punjab, indicating that drinking water samples in many areas across the province are unfit for human consumption [70].

### 3.4. Heavy Metal: Arsenic

**Figure 5:**
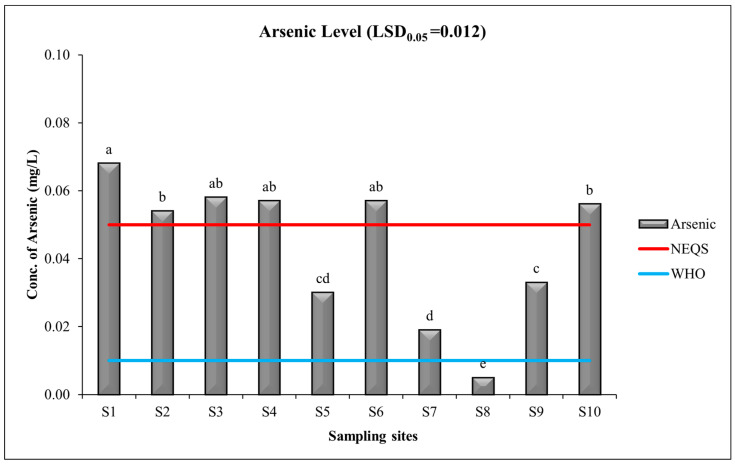
Concentration of arsenic in in water samples in different areas of Lahore. Mean values are given in the graphs. Horizontal lines show standard values for the parameter (red line represents NEQS and blue line represents WHO guideline). Letters (e.g., a, b and c) show that values are significantly different.

The average As level in drinking water samples was found to be 0.0437 mg/L, with minimum and maximum levels of 0.005 and 0.068 at S8 and S1, respectively. ANOVA results indicated that the levels of As in drinking water samples of sampling sites are significantly different. The following clusters were distinguished on the basis of the statistical analysis (LSD_0_._05_ = 0.012): a—0.068 (S1); b—0.054 (S2), 0.056 (S10); c—0.033 (S9); d—0.019 (S7); e—0.005 (S8); ab—0.058 (S3); 0.057 (S4); 0.057 (S6); and cd—0.03 (S4). The present study revealed a concerning situation regarding the presence of arsenic in the drinking water of selected urban areas of Lahore city. Water in six sites had concentrations of arsenic that were higher than those of the NEQS and WHO guidelines (Figure 5). Arsenic is a detrimental heavy metal that causes serious harm to ecological and human health [71,72]. The NEQS limit of arsenic is <0.05 mg/L. However, the concentration of arsenic is increasing day by day due to industrial processes and unchecked and uncontrolled anthropogenic activities [73]. The recent investigation in Lahore indicates that the levels of arsenic found are similar to those reported in other studies for Lahore [74,75]. According to the report, among the cities, Lahore is determined to be the most affected, while Muzaffarabad is considered the least affected. Analyzing the arsenic data in Lahore revealed that the water parameters in western Lahore, near the River Ravi were within safe limits. However, the remaining areas of Lahore exceed the permissible limit set by the World Health Organization (WHO), causing a significant population to face the serious issue of arsenic-contaminated drinking water.

### 3.5. Water Quality Index

**Figure 6:**
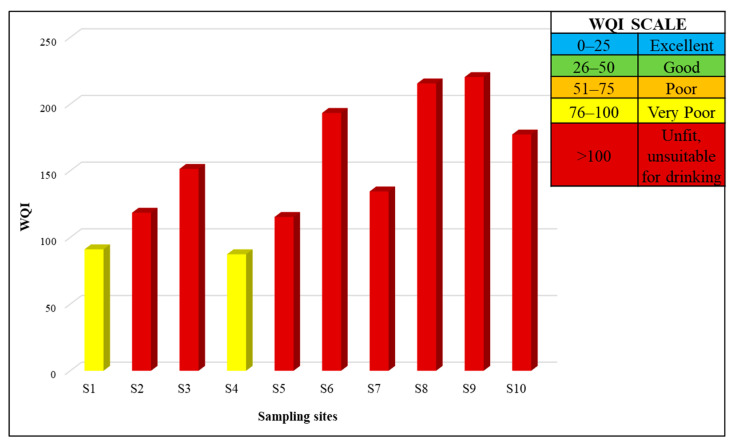
Water quality index of water samples in different areas of Lahore. WQI was estimated using mean values of different parameters. Red-colored bars show that water is unfit and unsuitable for drinking purposes. Yellow color bars shows that water has a very poor WQI.

Drinking water at S2, S3, S5, S6, S7, S8, S9 and S10 had a WQI of 118, 151, 115, 193, 134, 215, 220 and 177, respectively. These results make the water unfit and unsuitable for drinking purposes. The results showed that the water quality in selected areas of Lahore was very poor based on physico-chemical factors. The two areas at S1 and S4 had a WQI of 87 and 91, respectively. Water quality in these sites was characterized as very poor. It indicated that water in these areas had some physiochemical parameters that had higher levels of pollutants and posed a serious health threat to the residents of these areas, as shown in Figure 6.

In Pakistan, major cities including Islamabad, Rawalpindi, Lahore, Faisalabad and Karachi have 4000 patients of hepatitis due to the unsafe drinking water and the lack of treatment of water [76]. Hence, in developing countries, major diseases in children are caused due to the consumption of unfit drinking water. According to the survey of Pakistan National Conservation Strategy, in Pakistan 40% of transmissible diseases are linked to water. However, the actual status of water in mostly urban areas is declining rapidly in Pakistan. Due to groundwater contamination, the quality of water is declining steadily. However, in Pakistan almost 100 million diarrheal cases are reported annually [77]. According to the WQI model, 2.13, 6.38, 55.32, 22.34, and 13.83% of the water samples were excellent, good, bad, very poor, and unfit for drinking. Overall, the present study showed that the majority of the areas’ groundwater does not adhere to WHO standards. Therefore, there is a substantial risk to human health in the use of groundwater. The quality of the groundwater in New Karachi Town was found unfit for consumption [78]. According to the WQI, groundwater falls under the category of water quality that is unfit for drinking, indicating that proper treatment is necessary before its use.

### 3.6. Human Health Risk Assessment

#### 3.6.1. Chronic Risk

**Figure 7:**
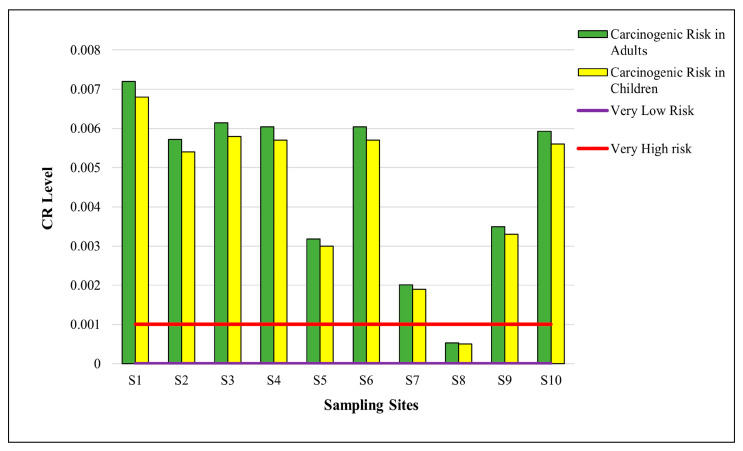
Carcinogenic risk of arsenic in water samples in different areas of Lahore. Mean values of parameters were used to estimate CR. Horizontal line shows standard value for the parameter (purple line represents very low cancer risk and red line represents very high cancer risk).

It is crucial to evaluate the potential health risks associated with the long-term ingestion of arsenic, as it is the most abundant element in the drinking water of the study area. This study focused solely on the ingestion pathway to evaluate the non-carcinogenic (HQ and HI) and carcinogenic health risks, as shown in Table 4 and Figure 7. The results indicated that all groups experienced an increase in HQ values. Additionally, the HQ values for As were greater than 1 for both children and adults at all sampling sites. It is found that the source of drinking water poses a significant lifelong health risk to all groups. To assess the non-carcinogenic effects of As, the HI was employed in the present study. Arsenic had a HI greater than 1 (USEPA standard), mostly due to the higher HQ value of arsenic. The maximum HQ was found in the drinking water of S1 (Chota Gaon Shahdara), i.e., 16 for adults and 15 for children. The HI findings indicated that residents of these areas experience a significant non-carcinogenic health risk across both age groups including children, and adults.

Residents of the study area may experience a number of non-carcinogenic health risks due to drinking unfit water on a regular basis. These health issues include nausea, abdominal pain, diarrhea, skin injuries, digestive and respiratory tract infections, liver damage, problems with the nervous, hematological, and cardiovascular systems, diabetes, problems with reproduction and hair loss, and neurological problems [79,80,81].

#### 3.6.2. Carcinogenic Risk Assessment

Figure 7 shows the computed CR results for both groups, namely children and adults. The average CR values were 4.37 (adults) and 4.60 × 10^−3^ (children) among every 10,000 residents of the study area. Cancer risk is associated with drinking water use. Between the two groups, adults had a higher CR than children did, as indicated by the mean value of CR.

The results of the present study, however, indicate that the CR value for both children and adults was higher than the USEPA’s (1 × 10^−6^ to 1 × 10^−4^). This indicates the possibility that children and adults may be at increased risk of cancer from consuming the arsenic-contaminated water in the study areas.

It was found that drinking water in the selected areas of Lahore city had a problem of contamination. This might be due to several reasons including the improper disposal of industrial and municipal effluents [82], deterioration of sewerage system, leakage of sewage and mixing with drinking water [13] and widespread application of fertilizers in the agriculture sector [60]. Arsenic contamination of groundwater has also been reported in other studies [71,83,84].

## 4. Conclusions

This study evaluated the quality of drinking water, contamination levels, and health risk assessment in selected urban areas of Lahore. The results showed that the drinking water in all selected sampling sites was unfit for human consumption according to the water quality index. Most sites had higher levels of arsenic, fluoride, TDS, residual chlorine, and *E. coli* than recommended by the NEQS and WHO guidelines. The WQI for most physico-chemical and microbial parameters indicated that drinking water was unsuitable for consumption (WQI > 100), except for that in Bhatti Gate (WQI = 87) and Chota Gaon Shahdra (WQI = 91). The human health risk assessment revealed that water posed a high risk to children and adults. The carcinogenic risk due to arsenic was also high for both adults (4.60) and children (4.37 × 10^−3^). Based on the findings of the present study, managers or decision makers should ensure the provision of safe drinking water by taking immediate action to address the contamination in these areas. This may involve implementing emergency measures, such as providing alternative sources of safe drinking water, until a long-term solution is in place. Therefore, responsible authorities can take practical measures to address the contamination issues and ensure the provision of safe and healthy drinking water to the residents of the selected urban areas in Lahore.

## Figures and Tables

**Table 1:** NEQS and WHO standards for different parameters of drinking water.

Sr. No.	Parameters	NEQS	WHO Guideline
1	Color	<15 TCU	15 TCU
2	Turbidity	<5 NTU	<5 NTU
3	pH	6.5–8.5	6.5–8.5
4	TDS	<1000	1000 mg/L
5	Nitrates	<50 mg/L	50 mg/L
6	Fluorides	<1.5 mg/L	1.5 mg/L
7	Residual Chlorine	0.2–0.5 mg/L	0.2 mg/L
8	Total Hardness	<500 mg/L	500 mg/L
9	Arsenic	<0.05 mg/L	0.01 mg/L
10	*E.coli*	0 CFU/100 mL	0 CFU/100 mL

**Table 2:** Sampling details: sample size, preservatives and holding time.

Parameter	Container	Mini. Sample Size	Preservative	Holding Time	Reference
*E. coli*	Sterile autoclave glass bottles	100 mL	<10	30 h	[36]
Fluoride	Plastic bottles	100 mL	<4	28 days	[37]
Nitrate	Plastic bottles	100 mL	<4	Immediate analysis	[36]
Residual chlorine	Glass bottles	500 mL	None	Immediate analysis	[37]
pH	Plastic bottles	50 mL	None	Analysis within 15 min	[36]
Turbidity	Plastic bottles	100 mL	<4	Immediate analysis	[37]
TDS	Plastic bottles	100 mL	<4	Immediate analysis	[36]
Arsenic	Plastic bottles	1000 mL	HNO_3_ to pH < 2	6 months	[36]

**Table 3:** Categories of water quality index: value, ratings and usage [32].

WQI Value	Water Quality Ratings	Usages
0–25	Excellent	Drinking, irrigation and industrial
26–50	Good	Domestic, irrigation and industrial
51–75	Poor	Irrigation
76–100	Very Poor	Restricted use for irrigation
>100	Unfit, unsuitable for drinking	Proper treatment required before use

**Table 4:** Non-carcinogenic risk according to chronic daily intake (*CDI*) and hazard quotient (HQ) in adults and children.

Site	Site Name	As (Mean Conc.)	Adults		Children	
			*CDI*	HQ	*CDI*	HQ
S1	Chota Gaon Shahdara	0.068	0.00480	16	0.004533	15
S2	Sant Nagar	0.054	0.00381	13	0.0036	12
S3	Bhutto Colony Shahdara	0.058	0.00409	14	0.003867	13
S4	Bhatti Gate	0.057	0.00402	13	0.0038	13
S5	Brendreth Road	0.03	0.00212	7	0.002	7
S6	Nishtar Colony	0.057	0.00402	13	0.0038	13
S7	Gajjumata	0.019	0.00134	4	0.001267	4
S8	Attari Saroba	0.005	0.00035	1	0.000333	1
S9	Tibba Kacha	0.033	0.00233	8	0.0022	7
S10	Islampura	0.056	0.00395	13	0.003733	12

## Data Availability

All the data are contained in the manuscript.
